# Expanding scope of Kirkpatrick model from training effectiveness review to evidence-informed prioritization management for cricothyroidotomy simulation

**DOI:** 10.1016/j.heliyon.2023.e18268

**Published:** 2023-07-25

**Authors:** Victor Kai-Lam Cheung, Nam-Hung Chia, Sze-Sze So, George Wing-Yiu Ng, Eric Hang-Kwong So

**Affiliations:** a Multi-Disciplinary Simulation & Skills Centre (MDSSC), Queen Elizabeth Hospital, Hong Kong Special Administrative Region; b Department of Neuroscience, Psychology and Behaviour, University of Leicester, UK; c Department of Surgery, Queen Elizabeth Hospital, Hong Kong Special Administrative Region; d Intensive Care Unit, Queen Elizabeth Hospital, Hong Kong Special Administrative Region; e Department of Anaesthesiology & Operating Theatre Services, Queen Elizabeth Hospital, Hong Kong Special Administrative Region

**Keywords:** Training effectiveness, Modified Kirkpatrick, Surgical Cricothyroidotomy, Medical simulation, Evidence-based management, Organizational change

## Abstract

Modified Kirkpatrick model has been adopted to evaluate training effectiveness by 6 categories, including activity accounting (training objectives/success in organization change) at Level-0, reaction (satisfaction) at Level-1, learning (acquisition of surgical airway skills) at Level-2, behavior (post-training change in personal strengths) at Level-3, result (organizational or clinical outcomes) at Level-4, and Return on Investment (ROI) or Expectation (ROE) (monetary and societal values following training and other quality and safety related measures) at Level-5. The purpose of this hospital-based prospective observational study was twofold: i) To evaluate potential impacts on monetary and societal values and successful organization change following implementation of advanced Cricothyroidotomy simulator and standardized curriculum in healthcare simulation training, ii) To inform decisions of resource allocation by reviewing overall values and prioritization strategies for i) general surgeon/emergency physician ii) with seniority >5 years and iii) prior porcine training experience based on findings at Kirkpatrick Level-0, Level-4, and Level-5. Seventy doctors and 10 nurses completed Cricothyroidotomy training and follow-up questionnaires within 2021/22. All training usability scoring measured by Scales of Emergency Surgical Airway Simulator (SESAS-17) achieved over 4 out of 5 (Level-4) with effects in favor of emergency physicians or general surgeons (*p* < .5), regardless of seniority and prior training experience. Success in organization change (Level-0) and cost-effectiveness (Level-5) were hypothetically established using theoretical framework of Gleicher's formula and Roger's Diffusion of Innovation Theory. Overall training effectiveness, in terms of advantage in usability, cost-benefits and successful organizational changes, provided sound evidence to support continuous investment of new curriculum and innovative simulator and “Surgeon-and-emergency-physician-first” policy when it comes to resources allocation strategies for Cricothyroidotomy training. [ACGME competencies: Practice Based Learning and Improvement, Systems Based Practice.]

## 1 Introduction

Evaluation of simulation training effectiveness has been positioning on a pivotal role in a wide range of service related to medical and occupational psychology from business to healthcare industries through 2028 [1]. Over a half century ago, Kirkpatrick has identified needs of developing an analytical framework to address the extent to which training programs contribute to learners’ development in job-related competencies, service quality and safety, and their influence on the community. Kirkpatrick [2] established the first widely adopted model in 4 levels, namely Reaction (subjective feeling or satisfaction of trainees), Learning (knowledge and skills acquired), Behavior (knowledge, skills or other personal qualities transferred), and Results (ultimate performance or measurable impact on the organization).

### 1.1 Comparison of training evaluation models

Further to development of the 4-level model, some researchers endeavored to expand the coverage of training impacts on higher level **(**Table 1**)**. Kaufman and Keller [3] made an attempt to add a new level on top of Kirkpatrick's model, addressing societal impacts on ecosystem of organization around the unit conducting medical education activities. This move made change of the model to cover benefits of training not only on target participants of the training (e.g., healthcare professionals) but also on high management of public hospital and the community (e.g., corporate image). Similar to Kaufman and Keller's [3], Phillips [4] expanded the level of model to the fifth one as well. However, Phillips tended to differentiate the Return on Investment (ROI) from performance outcomes in a more concrete and definable manner. ROI could be extrapolated by either quantifiable monetary values or unsolidified impacts other than money and specific training outcomes. The former one is theoretically stemmed from economics framework, which demonstrates cost-benefit relationship in value of investment (e.g., added benefit/avoided loss) of resources allocation on training process. The latter one is rather vague and unmaterialistic, such as improved trust by the community and stability in organizational structure and team morale. In response to Phillips' efforts, Kirkpatrick and Kirkpatrick [5] suggested modifying the term “ROI” to Return on Expectation (ROE), so that emphasis on monetary value would be combined with common interest of the community.

**Table 1:** Modified kirkpatrick model and other educational evaluation tool of training effectiveness.

Model\Phase	Planning and Design			Implementation	Just after Training		Post Training		
**Kirkpatirck (1959, 1995)**					1. Reaction	2. Learning	3. Behavior	4. Results	
**CIRO (Warr et al., 1970)**	1. Context	2. Input		3. Process		4. Product			
**Stufflebeam (1983); CIPP (Calvin, 1993)**	1. Context	2. Input			3. Reaction	4. Outcome			
**Brinkerhoff (1987)**		1. Goal Setting	2. Program Design	3. Program Implementation	4. Immediate Outcomes	5. Intermediate/Usage Outcomes	6. Impacts and Worth		
**Systems Approach (Bushnell, 1990)**		1. Inputs		2. Process	3. Outputs	4. Outcomes			
**Kraiger, Ford & Salas (1993)**					3. Affective	1. Cognitive	2. Skill-based		
**Kaufman & Keller (1994)**					1. Enabling and Reaction	2. Acquisition	3. Application	4. Organizational Outputs	5. Societal Outputs
**Holton (1996)**	1. Secondary Influences	2. Motivation Elements	3. Environment Elements			4. Outcomes	5. Ability/Enabling Elements		
**Molenda et al., (1996)**			**0. Activity Accounting**		1. Reaction	2. Learning	3. Transfer of Training	4. Business Impact	**5. Social Impact**
**Phillips (1997)**					1. Reaction/Planned Action	2. Learning	3. Applied Learning	4. Business Result	**5. ROI/ROE**
**Clark (2012)**					4. Motivation	3. Learning	2. Performance	1. Results/Impact	

Note. ROI = Return on Investment, ROE = Return on Expectation.

Modified Kirkpatrick model in this study included Levels from 0 to 5.

Level 0 --- Activity Accounting (Curriculum, Training Objectives, Success of Organization Change).

Level 1 --- Reaction (Satisfaction).

Level 2 --- Learning (Acquisition of Knowledge and Skills).

Level 3 --- Behavior (Personal Strengths/Knowledge and Skills Transfer).

Level 4 --- Results (Clinical Performance/Organizational Impacts).

Level 5 --- ROI/ROE (including Monetary and Societal Impacts).

From another angle, Clark [6] acknowledged the importance of elements mentioned in Kirkpatrick's model, whereas he claimed that success in effectiveness evaluation and course planning should depend on reverse sequence, starting with results to reaction which means using top-down approach instead of bottom-up approach. That being said, most researchers in educational training concerned about neither the sequence nor the relative weights of the levels because they often used it as an analytical framework as opposed to a guiding tool for course development [6].

### 1.2 Is training effectiveness in evaluation an outcome measures?

Different from those in mainstream training evaluation studies treating “evaluation outcomes” as post-training products, some researchers argued that training validity of evaluation outcomes should cover the entire system from overall planning to post-training outcomes **(**Table 1**)**. Warr and colleagues [7] proposed a model named “CIRO”. Starting with operation situation (training needs, objectives) in the Context and methodological design of Intervention (or Input), training effectiveness can be measured by subsequent Reaction (e.g., subjective feelings, or perceived changes in knowledge/skills) and Outcomes at immediate, intermediate, and ultimate level. Stufflebeam [8] criticized Kirkpatrick model in a similar way, emphasizing the importance of using evaluation as a tool not only for final reporting of the training effect but for continuous service enhancement from the outset. He suggested the CIPP model which covers Context (objectives and their relevance to organization cultures and service needs), Input (resources utilized and stage of training development), Process (feedback on training content, mode, and instructor), and Project evaluation (training outcomes addressed in 5-level model of Phillips).

Regardless of naming or abbreviations used, these models shared similar concepts with Kirkpatrick and Phillip's model, except their inclusion of elements in planning phase as training effectiveness. On top of Kirkpatrick's and Kaufman's 5-level Model, Molenda and colleagues [9], without emphasis on linear or hierarchical structure, added the sixth stratum, namely activity accounting (on training needs, sessions and number of participants, acceptance/ willingness for the training), at the beginning of their framework. This becomes one of the pillars of success in organization change in training simulator [[10], [11], [12]].

### 1.3 Modified Kirkpatrick model (Level-0): Evaluation on success in organization change

Beckhard [13] theorized that planned Intervention, a well-planned change can allow for a smooth transition in an organization, would be defined under certain conditions, namely i) planned (no band-aid or quick fix), ii) organization wide (relating to the entire system), iii) top down, iv) increased organization effectiveness and health, v) scientific mindset (evidence-based knowledge). Application of Gleicher's formula (in Dannemiller version) could help Industrial/Organizational (I/O) Psychologists predict or review whether the change could be implemented effectively [14]: Successful change would be foreseeable when effect of multiplicative factors (dissatisfaction level of current conditions, vision, and first step towards the vision) is greater than resistance (see “2.3.2.” for the formula and its manifestation).

### 1.4 Research questions, gaps and objectives

Here we set out two research questions.

1. What potential impacts have implementation of advanced Cricothyroidotomy simulator and standardized curriculum had on overall training effectiveness, in particular monetary and societal values and organization change, in healthcare simulation training for medical and nursing staff from trauma service in the Hospital Authority?
2. How does study findings inform decision of resources allocation for limited training quotas, in consideration of existing resource prioritization policy including i) general surgeon/emergency physician, ii) seniority >5 years, and iii) prior experience in training with porcine model)?

A research gap has long been identified in mainstream healthcare education studies regarding evaluation of training effectiveness: Hardly did researchers touch on behavioral change following training (Level-3), not to mention organizational outcomes (Level-4) nor cost-benefits (Level-5) [12,[15], [16], [17]]. To date, no academic paper has been identified to cover how innovative 3D-printed simulator as replacement [18,19] with standardized curriculum used to structurally assess training effectiveness on Level-4 or above, in particular ROI or cost-effectiveness analysis, in a reproducible manner [11,[19], [20], [21], [22]].

The study aims to evaluate post-training effect of emergency surgical airway based on variables taken as major criteria, namely specialty priority, seniority, and prior experience with porcine model. This is the first study applying modified Kirkpatrick model to assess training effectiveness, in particular Level-0, Level-4 and Level-5, in order to inform decision of hospital management in resources allocation, such as training quotas and target participants, continuation or discontinuation in training using new simulator and standard curriculum [23,24] (see Appendix 1–3).

Regarding practical consideration in organization impacts, high management would expect to examine whether the decision of prioritizing training quota for i) general surgeon or emergency physician, ii) seniority (>5 years in the specialty), and iii) prior experience in training with porcine model. It is postulated that.

• There would be successful organization change at Level-0 (Activity Accounting) with advancement in training simulator and standardized curriculum (Hypothesis 1).
• There were significant differences in all domains of training usability at Level-4 (Results) when comparing rating scores of participants with higher training priority than that with lower training priority (Hypothesis 2).
• There would be relative advantage of using new simulator in terms of cost-effectiveness at Level-5 (ROI) over other training modality (Hypothesis 3).

## 2 Methods

### 2.1 Design and procedure

This is a hospital-based review on effectiveness of surgical Cricothyroidotomy skills training under observational descriptive study design. Multi-Disciplinary Simulation and Skills Centre (MDSSC), a high-fidelity simulation training center located at the Queen Elizabeth Hospital, has been staffed by esteemed members with professional backgrounds in medicine and surgery, hospital administration, biomedical engineering, and occupational psychology. After passing through phases of implementation and evaluation, members further analyzed post-training data in order to facilitate annual review on organization change and prioritization policy of resources allocation in the hospital [15].

#### 2.1.1 Recruitment

Due to limited training quotas, all registrants were nominated by department heads or their delegate from 5 trauma centers governed by the Hospital Authority. Under this nomination system, neither sampling method nor randomization was performed during selection period between 2021 and 2022. To ensure foundation of life-saving knowledge and skills, all nominees have completed the Advanced Trauma Life Support (ATLS) Training as a pre-requisite of the surgical airway training. Given the higher training needs for emergency physicians and general surgeons [25], training quotas for healthcare professionals were assigned on fixed ratio by 2-tier priority.

• 1st Priority: 50 staff, including 35 general surgeons, and 15 emergency physicians
• 2nd Priority: 30 staff, including 6 neurosurgeons, 4 orthopedic surgeons, 10 anesthesiologists or intensivists, and 10 emergency nurses

Nominees who were i) at the age of 18 or below, ii) unable or reluctant to give informed consent prior to the training, or being failed to complete iii) the entire training or iv) post-training questionnaire would be excluded from the study.

#### 2.1.2 Ethical considerations

**Fig. 1:**
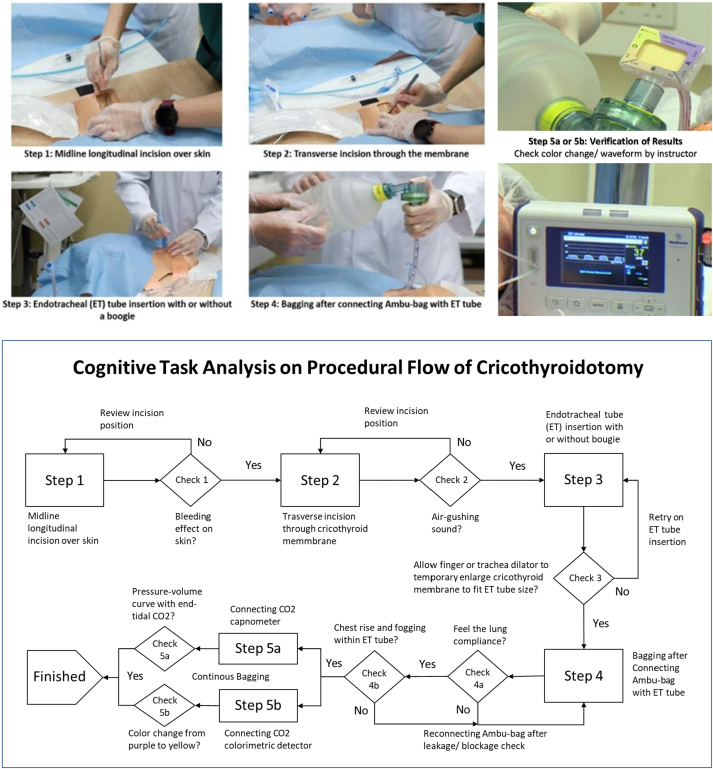
Visualization of 4-step (+ verification of results) procedures of cricothyroidotomy simulation.

Organization agreement for the training center and official ethical approval from the University Research Ethics Committee have been granted prior to data retrieval. All participants completed informed consent on confidentiality issues and use of data through electronic registration system on arrival [26]. De-identified information was retrieved by the principal investigator for aggregative analysis after the completion of 8 small-group training sessions based on standardized curriculum and procedures of Cricothyroidotomy (see Fig. 1 and Appendix 3).

### 2.2 Measurements --- SESAS-17

#### 2.2.1 Contents

The questionnaire, named “Scales of Emergency Surgical Airway Simulator (SESAS-17)”, was drafted by the principal investigator with professional qualification of Registered Test User (RQTU Occupational: Ability & Personality, Euro-test Level-2; British Psychological Society) and revised by workgroup members to enhance readability and understandability (see Supplementary 1 for full questionnaire of the SESAS-17). SESAS-17 consists of 17 rating questions, examining the extent to which the simulator achieved satisfactory usability for Kirkpatrick Level-4 (Results) and Level-5 (ROE) partially, in terms of.

• Design: Representation of genuine human anatomy
• Realism: Degree to mimic simulator characteristics, procedures, and function of Cricothyroidotomy when dealing with real patients (with “Physical”, “Procedural”, and “Functional” fidelity)
• Physical Fidelity: simulating tactile sensation on bagging
• Procedural Fidelity: simulating entire surgical airway steps and procedures
• Functional Fidelity: simulating functions of observable outcomes in each step (e.g., air leak, bleeding, chest rise)
• Functionality: Knowledge and skills acquisition, transfer to daily practice, and potential use for assessment
• Efficacy: Simplicity in individual practice
• Appraisal: Preferred modality compared to porcine model with satisfaction and recommendation to counterparts
• Safety: Low restriction and perceived risk of infection

All 5-point Likert scales ranged from 1 (Strongly disagree) to 5 (Strongly agree), and 3 (Neutral) as a mid-point. Responses at 4 (Agree) or 5 (Strongly agree) were counted as “positive responses”. Three questions, including 1 optional question and 2 open-ended questions were added at the back of SESAS-17 to explore raters’ experience in using pig and/or basic 3D-printed model in any surgical airway training, comments on simulator, and suggestions for improvement [27,28]. The survey was carried out online right after the Cricothyroidotomy training. On receiving designated QR code and relevant link for evaluation, participants were asked to complete the course evaluation and SESAS-17 with their own electronic devices (e.g., computer or smartphone) accordingly.

#### 2.2.2 Validation

Psychometrics were taken into account: i) Content validity: The extent to which workgroup experts show agreement in items relating to content of description, and ii) Internal consistency: The extent to which participants show consistency of responses across items on the integral set of questionnaires [29]. To minimize measurement biases, 50 volunteers who participated in previous surgical airway training were invited to rate on SESAS-17 after validating questions by 6 workgroup members, resulting in satisfactory inter-item consistency (*Cronbach's α* = .92), and content validity quantified by average of summary content validity index (*S-CVI/Ave* = 0.94) and scale content validity index with universal agreement (*S-CVI/UA* = 0.82) [30] **(**Table 2**)**.

**Table 2:** Content validity indexes of scales of emergency surgical airway simulator (SESAS-17).

Item	Domains	Item Description	Workgroup Raters						Agreement no.	I_CVI
			NC	GN	ES	VC	ML	IC		
01.	1	Representation of genuine human anatomy	**✓**	**✓**	**✓**	**✓**	**✓**	**✓**	6	1
02.	2	Physical Fidelity: Tactile sensation on bagging	**✓**	**✓**	**✓**	**✓**	**✓**	**✓**	6	1
03.	2	Procedural Fidelity: Mimicking entire surgical airway procedure	**✓**	**✓**	**✓**	**✓**	**✓**	**✓**	6	1
04.	2	Functional Fidelity: “Air leak” upon incision of cricothyroid membrane	**✓**	**✓**	**✓**	**✓**	**✓**	**✓**	6	1
05.	2	Functional Fidelity: “Bleeding” after incision of skin	**✓**	**✓**	**✓**	**✓**	**✓**	**✓**	6	1
06.	2	Functional Fidelity: Chest rise and fogging of endotracheal tube	**✓**	**✓**	**✗**	**✓**	**✓**	**✓**	5	.83
07.	3	Acquisition of knowledge and skills	**✓**	**✓**	**✓**	**✓**	**✓**	**✓**	6	1
08.	3	Transition from practice to daily operation	**✓**	**✓**	**✓**	**✓**	**✓**	**✓**	6	1
09.	3	Performance assessment	**✗**	**✓**	**✗**	**✗**	**✓**	**✓**	**3**	.50
10.	4	Simplicity in practice	**✓**	**✓**	**✓**	**✓**	**✓**	**✓**	6	1
11.	4	Independent use	**✓**	**✗**	**✗**	**✓**	**✓**	**✓**	**4**	.67
12.	5	Satisfaction with model	**✓**	**✓**	**✓**	**✓**	**✓**	**✓**	6	1
13.	5	Recommendation to counterparts	**✓**	**✓**	**✓**	**✓**	**✓**	**✓**	6	1
14.	6	Preference on 3D printed model (No pig neck)	**✓**	**✓**	**✓**	**✓**	**✓**	**✓**	6	1
15.	6	Preference on advanced simulator (Not 3D printed model alone)	**✓**	**✓**	**✓**	**✓**	**✓**	**✓**	6	1
16.	7	Perceived lower risk of infection	**✓**	**✓**	**✓**	**✓**	**✓**	**✓**	6	1
17.	7	Low restriction in practice	**✓**	**✓**	**✓**	**✓**	**✓**	**✓**	6	1

**Note.** Domains are: 1. Design, 2. Realism (Physical, Procedural, and Functional Fidelity), 3. Functionality, 4. Efficacy, 5. Appraisal, 6. Preference, 7. Safety. Item 9, 10, and 11 were modified for content specification before use in trial and training sessions.

- I_CVI = Content validity of each item = no. of rater agreed on item/total no. of all rater.

- S-CVI/Ave = Average of summary content validity index = sum of content validity of all items/total no. of items = 0.941.

- S-CVI/UA = Scale Content Validity index with universal agreement = no. of items on universal agreement/total no. of items = 0.823.

### 2.3 Data analysis

#### 2.3.1 Statistical methods

Descriptive Statistics on demographic information, including type of professional/specialty, gender, training priority (1st or 2nd), seniority (>5 years or not), and prior experience with pig model, were reported by counts and percentages.

To investigate between-group differences (Partially Kirkpatrick's Level-4 Results), participants were stratified into 2 subgroups as independent variable by “Training priority”: i) General surgeon & Emergency physician, ii) Other doctors/nurses (see “2.1.1. Recruitment”). Independent sample t-tests were used for between-group comparison of population means of the 6 domains in the SESAS-17 as dependent variables using SPSS software (ver. 25, IBM Corp., 2017). All significant levels of Alpha were set at 0.05 (two-way), if not otherwise indicated. The same method would be applied to other independent variables, namely “Seniority” (>5 years or not), and “Prior experience in porcine model” (Yes/No).

#### 2.3.2 Hypothetical analysis on activity accounting (Level-0) and organizational impacts (Level-4 and 5)

Combined with Molenda et al. [9] and Phillip's [4] expansion, a modified Kirkpatrick's model was adopted partially to evaluate training effectiveness and organizational impact.

• Level-0: Activity Accounting: Training needs and acceptance of design, planning and implementation
• Level-4: Result: Anticipated operational impacts, or clinical outcomes in an organization
• Level-5: Return on Investment (ROI)/Return on Expectation (ROE), or cost-effectiveness analysis (added or avoided cost) or influence on the society

Results of Level-0 were supported by standardized training simulator, curriculum aligned with internal standard, procedure and workflow governed by hierarchical task analysis, and evaluation tool validated by workgroup members [13,14]. In order to evaluate on organizational change, a formula was used for quick concept calculation:

Successful Change = D*V*F > R Or = A*V*F > IC + EC

where D is “dissatisfaction level of the current condition”, V is “Vision”, F is “First step towards the vision”, and R is “Resistance” to change. When all factors on the left of the formula shows high in strengths, resistance could no longer stop the change happens. Based on Herzberg's Motivation-Hygiene Theory [31], a two-factor approach on standing both proactive and reactive sides contributing to workplace motivation (e.g., achievement/advancement, self-recognition, and accountability) and satisfaction (e.g., organization policy, security, and relationship), Mirza [32] reframed the model positively by replacing D (Dissatisfaction) by A (Aligned realization for motivational change), and considering Resistance (R) as IC (Internal challenges) and EC (External challenges).

Level-4 was evaluated with participants’ rating on overall performance and between-group comparison in all SESAS-17 domains. Level-5 would be explored on weighing development cost of simulator and its benefits to stakeholders as well as the organization (ROI) or to the society (ROE). Based on hypothetical numbers (e.g., numbers of patients for Cricothyroidotomy and survival rate) from literatures [33], three formulates were applied for cost-effectiveness analysis as follows.

• Cost-avoidance Outcomes = Additional costs - Cost of intervention
• Cost-benefit Analysis (CBA) = Saved ICU medical burden/Intervention cost >1
• Return on Investment (ROI) = (Saved ICU medical burden - Intervention cost)/Intervention cost >0

Organizational impact for evidence-informed decision-making process on resources allocation would be elaborated following the discussion of usability under the framework of Rogers’ Diffusion of Innovations Theory [34] in the “4. Discussion”.

## 3 Results

### 3.1 Level-0 (Activity accounting)

Followed by fixed quota nomination, 35 general surgeons (43.8%) and 15 emergency physicians (18.7%) were recruited as the first priority (*N* = 50, 62.5%), while 10 anesthesiologists or intensivists (12.5%), 6 neurosurgeons (7.5%), 4 orthopedic surgeons (5%), as well as 10 emergency nurses (12.5%) were recruited as the second priority (*N* = 30, 37.5%) **(**Table 3**)**.

**Table 3:** Demographic characteristics of participants in cricothyroidotomy training.

Characteristics (N = 80)		Counts	(%)
**Discipline**	*General Surgeon*	35	43.8
	*Emergency Physician*	15	18.7
	*Emergency Nurse*	10	12.5
	*Neurosurgeon*	6	7.5
	*Anesthesiologist/Intensivist*	10	12.5
	*Orthopedic Surgeon*	4	5
			
**Priority**	*First Priority*	50	62.5
	*Second Priority*	30	37.5
**Gender**	*Female*	38	47.5
	*Male*	42	52.5
**Seniority**	*1–5*	40	50
	*6–10*	26	32.5
	*11–15*	8	10
	*>16*	6	7.5
**Prior Training Experience with Porcine model**	*No*	35	43.7
	*Yes*	45	56.3

To re-address the formula of organization change:

Successful Change = Dissatisfaction*Vision*First Step > Internal Challenges + External Challenges

With all positive results from participants and satisfactory outcomes in overall scores, successful change of replacing animal model by newly invented simulator has been resulted from strong needs for change, clear vision and goals, capability to change, and last but not the least, concrete first step. Since all factors in the left were high while the factors (Resistance) on the right were extremely low, the likelihood of success in organization change was extremely high.

### 3.2 Level-4 (Results)

In order to evaluate the results from resource allocation policy, independent sample t-tests were used to compare between-group difference in all domains of SESAS-17 of different pairs of independent variables, namely i) first-second priority, ii) seniority, and iii) prior experience in porcine model.

#### 3.2.1 First priority v.s. Second priority

When stratified by priority level, domain scores of the first priority and the second priority ranged from 4.72 to 4.93 and 4.40 to 4.62, respectively. Using independent sample t-tests, statistically significant differences were found between two priority group for design, *t(78)=*2.43, and functionality, *t(78)=*2.58 at *p*<.05 level, and for realism, *t(78)=*4.78, efficacy, *t(78)=*2.75, appraisal, *t(78)=*2.71, and safety, *t(78)=*3.14 at *p*<.01 level **(**Table 4**)**.

**Table 4:** Comparing participant rating on SESAS-17 between 2 priority levels.

SESAS-17 Usability Domains	All Participants (N = 80) M ± SD	1st Priority (N = 50) M ± SD	2nd Priority (N = 30) M ± SD	Between-group P-value
**Design**	4.60 ± .59	4.72 ± .57	4.40 ± .56	.017
**Realism**	4.73 ± .38	4.88 ± .22	4.47 ± .44	.000
*Physical Fidelity*	4.74 ± .47	4.92 ± .27	4.43 ± .57	.000
*Procedural Fidelity*	4.66 ± .48	4.80 + .40	4.43 ± .50	.001
*Functional Fidelity*	4.78 ± .37	4.93 + .25	4.53 ± .42	.000
**Functionality**	4.76 ± .41	4.85 ± .34	4.62 ± .47	.021
**Efficacy**	4.67 ± .46	4.78 ± .41	4.48 ± .50	.008
**Appraisal**	4.74 ± .44	4.85 ± .35	4.57 ± .50	.01
**Safety**	4.71 ± .43	4.83 + .37	4.52 + .46	.003

Note. N = Valid number of participants; M = Mean; SD = Standard Deviation.

#### 3.2.2 Basic trainee v.s. Higher trainee/ Senior staff

With the cutoff of 5 in year of clinical experience, participants were divided into two even groups by happenstance for further analysis **(**Table 5**)**. Comparing participant rating on usability of simulator, no statistically significant difference was found in most domains of SESAS-17, except realism at *p*<.05 level, in particular physical fidelity at *p*<.01 level.

**Table 5:** Comparing participant rating on SESAS-17 between 2 seniority groups (cut off = 5 Years).

SESAS Usability Domains	Low Seniority (N = 40) M ± SD	High Seniority (N = 40) M ± SD	Between-group P-value
**Design**	4.70 ± .46	4.50 ± .68	.129
**Realism**	4.81 ± .30	4.64 ± .43	.039
*Physical Fidelity*	4.88 ± .33	4.60 ± .55	.008
*Procedural Fidelity*	4.73 ± .45	4.60 ± .50	.243
*Functional Fidelity*	4.84 ± .32	4.72 ± .41	.133
**Functionality**	4.81 ± .39	4.71 ± .42	.273
**Efficacy**	4.74 ± .44	4.60 ± .48	.186
**Appraisal**	4.80 ± .41	4.69 ± .46	.251
**Safety**	4.76 ± .42	4.66 ± .44	.306

Note. N = Valid number of participants; M = Mean; SD = Standard Deviation.

Simulator – Realism: Cohen's d = 0.472; 95% CI = 0.026 - 0.914.

→Simulator – Physical fidelity: Cohen's d = 0.608; 95% CI = 0.157–1.054.

#### 3.2.3 Prior experience in pig model v.s. No experience in pig model

Of 80 participants, 45 (56%) had experience in training with pig model, either live porcine or partial neck of animal tissues **(**Table 6**)**. For perceived usability of the new simulator and training effectiveness, those without such experience (44%) scored higher than their counterparts in almost all domains, except realism (specifically physical and functional fidelity), training design, instructor feedback, and mental preparedness with comparatively slight drop. Even so, no statistically significant results were found between groups at *p*<.05 level when applying independent sample t-tests for analysis, regardless of any domain in SESAS-17.

**Table 6:** Comparing Participant Rating on Usability Domains of SESAS-17 between Those with/without Prior Experience in Porcine Model.

**SESAS Usability Domains**	No Pig Model Experience (N = 35) M ± SD	Pig Model Experience (N = 45) M ± SD	Between-group P-value
**Design**	4.69 ± .53	4.53 ± .63	.252
**Realism**	4.72 ± .40	4.73 ± .37	.854
*Physical Fidelity*	4.66 ± .48	4.80 ± .46	.183
*Procedural Fidelity*	4.74 ± .44	4.60 ± .50	.179
*Functional Fidelity*	4.75 ± .41	4.80 ± .34	.572
**Functionality**	4.81 ± .39	4.72 ± .42	.317
**Efficacy**	4.70 ± .46	4.64 ± .47	.598
**Appraisal**	4.74 ± .44	4.74 ± .43	.987
**Safety**	4.76 ± .41	4.68 ± .45	.421

Note. N = Valid number of participants; M = Mean; SD = Standard Deviation.

### 3.3 Level-5 (ROI)

Estimated by worldwide annual statistics of prevalence rate at 0.03 per 10,000 persons, about 24 persons would require Cricothyroidotomy in HKSAR [33]. Given the post-Cricothyroidotomy survival rate was up to 75%, the estimated cost of failure of Cricothyroidotomy would be 18 human lives, or over 8 M per month when all of them suffered from severe anoxia brain injuries and on mechanic ventilators in the Intensive Care Unit (ICU) [35] **(**Table 7**)**.

**Table 7:** Cost-effectiveness analysis on simulation as intervention.

Category	Unit Cost (HKD)	Unit Cost (USD)	Operation Cost of Simulation with 80 trainee/ year (HKD)	Cost-avoidance Outcome (HKD)	Cost-benefit Analysis (CBA >1)	Return on Investment (ROI >0)
Emergency Surgical Airway Simulator (ESAS)	630	80	50,400	7.95 M	158.7	157.7
Live Porcine	3,500 (17,500 shared by 5 trainees)	440	280,000	7.72 M	28.6	27.6
Human Cadaver	7,500 (30,000 shared by 4 trainees)	950	600,000	7.40 M	13.3	12.3

Note. Estimated additional costs of ICU in survivor of anoxic brain injury following unsuccessful surgical Cricothyroidotomy for 1 month = 18 * 14,800 * 30 = 8 M.

Formula:

- Cost-avoidance Outcomes = Additional costs - Cost of intervention.

- Cost-benefit Analysis (CBA) = Saved ICU medical burden/Intervention cost > 1.

- Return on Investment (ROI) = (Saved ICU medical burden - Intervention cost)/Intervention cost > 0.

## 4 Discussion

### 4.1 Result summary of training effectiveness using modified Kirkpatrick's models

Overall training effectiveness (and impacts) of Cricothyroidotomy sessions were positively demonstrated at all Kirkpatrick Levels covered in this study.

#### 4.1.1 Level-0 (Activity accounting)

Hypothesis 1 is supported by the result that advancement in training with standard curriculum and new simulator can lead to successful organization change (Level-0: Activity Accounting). Learning objectives mentioned in Appendix 3 were met. All participants demonstrated skills and knowledge acquisition of Cricothyroidotomy by increasing awareness of “Cannot Intubate Cannot Oxygenate” (CICO) condition, site applying the procedure, and international standards (i.e. 2013 CAFG and 2015 DAS) by the end of the training [36,37]. Success in organization change was evaluable with Mirza's formula [32]. Course directors' dissatisfaction on applying animal model in current surgical airway training or strong motivation on innovative approach (high D or A), organization vision on continuous improvement in achieving healthcare excellence through simulation (high V), inventing a simulator using 3D-printing and innovative approach in multi-disciplinary team as the first step (high F) outweighed the resistance to change which has been minimized by all-level communication (low R, IC, and EC). Further discussion on the review of the significant effect of using new training curriculum and simulator will be covered in Discussion 4.2.

#### 4.1.2 Level-4 (Results)

Hypothesis 2 is supported by the results of how training effects can be translational to positive organizational impacts. Overall quality and safety in patient care reached high international standard. Regarding clinical outcomes, no patient died from medical incidents due to human errors in task, untrained personnel, delayed decision or operation time (>2 min) in Cricothyroidotomy. Regarding operational impacts, unified and standardized emergency airway management practice was utilized in not only corporate training but also hospital protocol and system workflow. Regarding organizational impacts, the study provided evidence to support decisions of ii) organization change in replacing traditional pig model by newly developed simulator for better usability and training effectiveness; and ii) prioritization policy of training resource that allocating training quotas to general surgeons and emergency physicians as the 1st priority, regardless of seniority (over 5-year experience in respective specialty) or prior experience in training with porcine model.

#### 4.1.3 Level-5 (ROI/ ROE)

Hypothesis 3 Regarding monetary and societal values, is supported by the findings. Benefits to hospital high management or public medical system could be analyzed by cost-effectiveness analysis based on full economics evaluation on “avoided costs” [10,38]. Simulation training experience may reduce decision time and operation time for successful surgical airway management and intubation within 5 min to prevent irreversible outcomes, either anoxic brain injury or death [39]. Furthermore, training values as “Return on Expectation” will be discussed in the next session based on Diffusion of Innovation Theory [34].

### 4.2 Elaboration on organization impact using rogers’ diffusion of innovation theory

#### 4.2.1 Relative advantage

Relative advantages represent the distinctive features of the new simulator, such as costs and innovation stood out from the market available product. Concept of “Costs” or “profitability” could be evaluated by partial or full economics on dimensions of number alternatives being considered/ costs per outcomes [40]. Irrespective of functional role of stakeholders (e.g., administrator/ clinical provider/ patient/ accrediting bodies/learner/societal …) or influence in time points or cultural background, monetary costs would be considered primarily. After ruling out “up-front” (e.g., infrastructure or center set-up fee) or “sunk” costs for existing service (e.g., simulation equipment, maintenance costs, space utilization), the production cost for each participant was around 80 USD only. When it comes to cost-effectiveness, the new simulator is over 12 times and 5 times of Live porcine model and human cadaver, respectively (Table 7).

Comparing with existing simulator composed with live tissue from pig or corpse, the simulator had no hygiene and logistics (transfer/disposal/decomposing during training) concerns, actualizing tactile sensation and human physiology superior to traditionally available low-fidelity part-task trainer for basic landmark identification and incision practice. The greatest breakthrough superior to market available simulator, was that the fiction on ET tube insertion was low when applying multiple 3D-printing materials (with hard and soft textures in designated parts) with injection molding technology [30,41]. Depending on training objectives, realism could be enhanced by combining both animal tissues and innovative technology, connecting the simulator with monitor showing vital signs (e.g., pulse, BP, SPO2, end tidal CO2), and even applying ultrasonography on neck area [42].

#### 4.2.2 Compatibility

Developing Cricothyroidotomy simulator fitted with the vision and missions of hospital high management. Depending on training purposes and resources, the simulator could be used alone for skill-based practice or assessment in surgical airway skill training or educational purpose to visualize anatomical landmarks. When combined with other training modalities, “hybrid simulation” allows trainee to handle CICO condition with another standardized patient for both technical skills on behavior domain of Cricothyroidotomy and communication skills on affect domain, for training and/or assessment purpose.

#### 4.2.3 Complexity

Participants and instructors acknowledged that the simulator was easy to use and could work without technical support. Administrators found that the simulator was barrier-free in management as it is movable, with low restriction in practice and risk of infection control and physical as well as psychological safety. After receiving professional guidance through formal Cricothyroidotomy training, participants may consider deliberate practice for skills transfer and retention in a long term [20,23].

#### 4.2.4 Trialability and observability

With the experience in conducting this study, detailed description and standard steps and procedures from how to develop the simulator to how to operate training as well as to evaluate usability of simulator and the training effectiveness could place a solid foundation to put plans into pilot test and further sessions. The degrees to which the use of simulator showed observable effects on successful operation were excellent. Aligned with learning outcomes, the measurable outcomes at the endpoint were clear. Through overt observation, instructors verified the effect of Cricothyroidotomy by connecting the simulator with capnometer or colorimetric detector of Carbon Dioxide (CO2) to ensure that the simulated airway and breathing functions was intact **(see** Fig. 1**)**.

### 4.3 Ultimate goal: Review on prioritization policy of resources allocation

There is no one-size-fit-all approach that could be applied directly to inform every decision of organizational change and prioritization policy of resources in hospital management. Consistent with training effectiveness model from Kaufman & Keller [3] and Stufflebeam [8], Scientific values and society impact following resources prioritization policy might be examined through research process [38,43,44]. When perceived fairness in accountability and transparency were continuously improved, evidence-based practice could inform decisions of resource prioritization in healthcare service [[45], [46], [47], [48]].

Same as Rojo et al. [11,12], this study employed innovation technology for frontline workers for organizational change in system reengineering and patient safety. Rather than being treated as a theoretical mechanism, structured evaluation on training effectiveness using modified Kirkpatrick model could benefit an organization from determining whether missions or key objectives have been achieved (e.g., number of trainees felt satisfied with the training, and completed training with proof of acquisition of surgical airway procedures at international standard). It also allowed policymaker in hospital executive management to make evidence-informed decision, such as i) prioritization of target participants for mandatory training under resources constraints (in this case training quota), ii) continuation or termination of funding support to the training, and iii) quality assurance and control measures for simulation training.

As a result, positive changes at all levels in modified Kirkpatrick model as a proof hospital management would entail less risk on investment. Under extremely high constraints on readily resources, such as training places with limited pool of qualified instructors and rooms for potential participants to be trained under protected hours during work, reviewing on existing resource allocation and prioritization policy in staff nomination with existing literature and current research findings deemed necessary.

#### 4.3.1 Pre-requisite: Advanced Trauma Life Support (ATLS) training

According to Demirel et al. [49], participants with ATLS training or equivalent record would show higher training effectiveness, in terms of time and accuracy (with confidence) to complete: 14s (or 18%) faster and 24% higher accuracy on incision over skin and cricothyroid membrane. As a mandate training of all medical and nursing staff in trauma management, the steering committee decided to keep this criterion as a pre-requisite of the advanced surgical trauma course.

#### 4.3.2 First priority v.s. Second priority

In line with the study result, priority policy for general surgeon and A&E physician should keep status quo. Surgeon and A&E physician in first priority outweighed other participants in all domains in usability and training effectiveness in this study, and personal strengths (except “assertiveness”) in another study of our team [15]. It is speculated that, comparing with scenario-based inter-professional simulation training, the training nature with emphasis on specific knowledge and skills acquisition (e.g., Cricothyroidotomy procedures) made team-based element, such as assertiveness, a less relevant attribute to grow using the simulator as sole training modality. Fulfulment of goal-directed objectives neccessitates right options for mode of teaching and type of simulator prior to implementation of the training.

#### 4.3.3 Seniority and prior experience in porcine model

Seniority and prior training experience with porcine model had no statistically significant effects on usability, training effectiveness, and personal strengths [17,19,50]. Despite preferences of junior staff on new simulator and relatively low learning effect for those with pig dissection experience, they were not significant enough for being considered in resources prioritization.

### 4.4 Strengths and limitations

This is the first evaluative review on effectiveness of Cricothyroidotomy training using new curriculum and simulator under modified Kirkpatrick model, showing feature of research initiatives and applicability of innovations as an opportunity to inform decision-making of organization change and prioritization policy of resources allocation. Nevertheless, several limitations have been identified.

#### 4.4.1 Market popularity

This is an essential topic with small market niche. Geographically, crisis preparedness for low prevalent condition requiring surgical Cricothyroidotomy would be much more ubiquitous in the US and some European countries, including inter alia Denmark and Spain, with the input from I/O Psychologists at business consultant/ managerial levels in medical education and simulation center management.

#### 4.4.2 Cost analysis

This is an exemplary study to close the research gap of cost-effectiveness for medical simulation [40]. Without available statistics in hospital server or annual report, the author could only make estimation using statistics from research. Avoided cost was based on hypothetical estimation without considering economic inflation, availability of ward beds, and variation in length of stay or severity of possible outcomes. When cost-effectiveness analysis was based on existing resources, the cost-benefit ratio may be exaggerated. Technically, replicating this study required not only standardized workflows but also inter-professional team with relevant expertise and experienced instructor at the rank of consultant surgeon. Therefore, exclusion of sunk and up-front costs of existing facilities and manpower resources would be feasible to high-fidelity simulation center at comparable level only.

#### 4.4.3 Restriction in research methods and data analysis

Arbitrary cut-off of 5 in seniority of participants was due to i) most basic trainees would become higher trainees after 4 years, ii) nurse would be more skillful to handle cases with doctor without mentorship, iii) more balanced sample size in two groups. However, it limited the choice of methods of statistical analysis (e.g., ANOVA requires 3 or more levels of independent variables), making multi-variate or post-hoc test for further analysis impossible.

Comparing the training effect and perceived usability of “simulator” by groups with or without “prior experience in pig model” might not completely equal to that in clinical studies, or RCT. Level of study was weakened when there was no control group nor comparison with alternative intervention arm (e.g., live porcine model, human cadaver model... etc). Without control and standardization in prior training workflow and quality, so-called “prior experience in training with porcine model” varies by intensity, type, and level of involvement in training. Irrespective of costs and availability of wet lab in external university-affiliated medical center, customs clearance from overseas company may result in delayed transportation during COVID-19 pandemic. When it comes to costs, feasibility of training items, and infection control, choice of using live pig as another intervention arm was not practical at the moment.

### 4.5 Conclusion

This study filled part of the research gap where most studies in training effectiveness fell into categories regarding training satisfaction and acquisition of skills and knowledge at Kirkpatrick Level-1 and Level-2 only. It was a breakthrough to examine potential impacts on monetary and societal values at Level-4 and Level-5 and successful organization change in healthcare simulation training at Level-0. Promising evidence from the study informed healthcare practitioners and administrator of how organization change from traditional live porcine model to simulator using advanced 3D-printing technology and innovative approach for Cricothyroidotomy training enhanced overall training effectiveness, in particular potential cost-benefit and organizational impacts. Beyond hypothetical values, usability of newly developed simulator evaluated by psychometrically sound measurement demonstrated practical values on reviewing resource allocation strategies. Based on study findings, priority of training quota should be given to general surgeons and emergency physicians regardless of seniority and prior experience in training with porcine model. It is recommended that evidence-based practice with analytic framework, such as modified Kirkpatrick's model, Gleicher's formula, and Rogers' Diffusion of Innovations Theory, could be incorporated into existing quality enhancement framework in healthcare simulation management. Beyond applying the findings to inform management decision of effective resource allocation, further research may explore whether other innovative technology, such as virtual reality, would add practical as well as educational value to Cricothyroidotomy simulation training and assessment under the modified Kirkpatrick model.

## Patent

Dr. N H Chia is one of the owners of Hong Kong Patent for “Emergency Surgical Airway Surgical Simulation Device” (#30041806).

## Funding

The authors did not receive any specific grant from funding agencies in the public, commercial, or not-for-profit sectors.

## Ethical statement

This study was supported by the Research Ethics Committee of the University of Leicester (Reference no.: #29837) with organization approval from the Multi-Disciplinary Simulation and Skills Centre, Queen Elizabeth Hospital.

## Author contribution statement

Victor Kai-Lam Cheung, Nam-Hung Chia: Conceived and designed the experiments; Performed the experiments; Analyzed and interpreted the data; Contributed reagents, materials, analysis tools or data; Wrote the paper.

Sze-Sze So: Conceived and designed the experiments; Analyzed and interpreted the data; Contributed reagents, materials, analysis tools or data.

George Wing-Yiu Ng, Eric Hang-Kwong So: Conceived and designed the experiments; Contributed reagents, materials, analysis tools or data.

## Data availability statement

Data will be made available on request.

## Declaration of competing interest

The authors declare that they have no known competing financial interests or personal relationships that could have appeared to influence the work reported in this paper.
