# Supplementary material for: Expanding scope of Kirkpatrick model from training effectiveness review to evidence-informed prioritization management for cricothyroidotomy simulation
Source: Heliyon. 2023 Jul 25;9(8):e18268. doi: 10.1016/j.heliyon.2023.e18268 (PMC10407669; doi:10.1016/j.heliyon.2023.e18268)
Supplement: Multimedia component 1 [file mmc1.pdf]

APPENDIXES

Appendix 1. Formulation of Training Framework of Surgical Airway Model

Categories	Old	New
Mode of Governance	Steering Committee of Advanced Surgical Trauma Course (ASTC)	Steering Committee of ASTC + Taskforce of Emergency Surgical Airway Training
Training Modality Level	low-fidelity Skill-based simulation	High-fidelity Skill-based simulation
Training Tools	Live porcine model/ Animal tissue	Simulator, applying 3D-printing technology and innovation with silicone for “skin”, tagaderm blood pad, alcohol and warm bag.
Trainer-Trainee Ratio	1:6	1:5
Maximum Training Capacity	36	80
Training Curriculum	Suggested training module<br>Based on experience of instructor	Standard international standard<br>Standard procedure and criteria of competence
Training Versatility	Training in wet lab and under guidance by qualified instructor	Initial training in any rooms under instruction; possible opportunities for deliberate practice
Manpower Required	Consultant surgeon + Technician	Initial training: Consultant surgeon + Technician<br>Deliberate practice: Trainee alone (± Technician)
Unit Cost/ Trainee (HKD)	3,500 – 7,500	630
Monetary Advantage	N/A	Used less than 20% cost of old framework to train over 2 times of trainees
Quality Assurance	N/A	Regularly assessed by Usability, Training effectiveness, and personal strengths using Simulation Training Course Evaluation and the SESAS-17
