# Supplementary material for: Expanding scope of Kirkpatrick model from training effectiveness review to evidence-informed prioritization management for cricothyroidotomy simulation
Source: Heliyon. 2023 Jul 25;9(8):e18268. doi: 10.1016/j.heliyon.2023.e18268 (PMC10407669; doi:10.1016/j.heliyon.2023.e18268)
Supplement: Multimedia component 2 [file mmc2.pdf]

Appendix 2. Setup of Emergency Surgical Airway Simulator (ESAS)

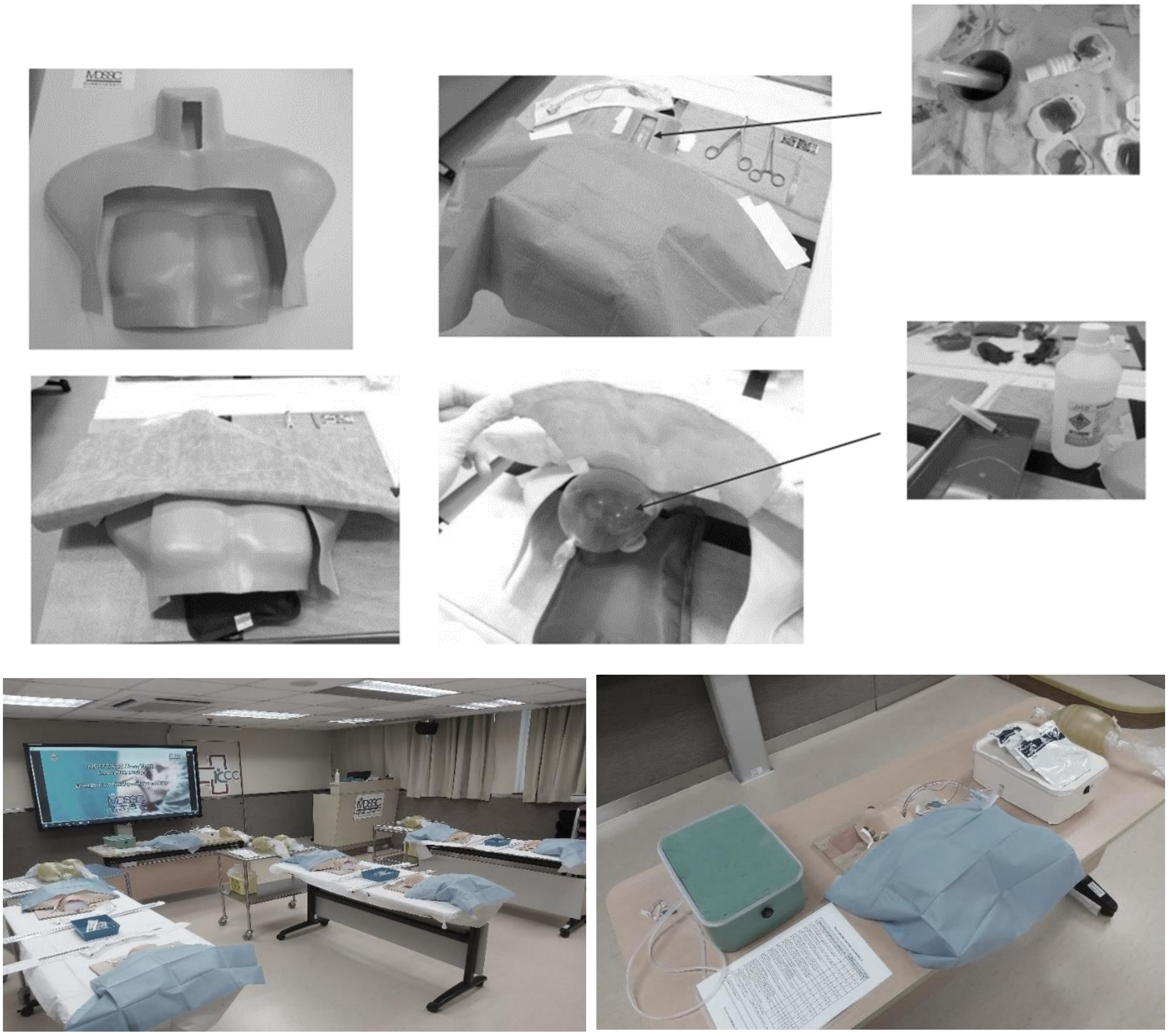

<u>Level of fidelity with Examples</u>

- **Low fidelity:** use real “pig neck” or low technology model dissimilar to human anatomical structure or ratio
- **Medium fidelity:** use basic “3D printed larynx-trachea model” with 1:1 human anatomical structure (for deliberate practice on skills with tactile sensation); or virtual reality program (for strengthening memory of surgical procedure)
- **High fidelity:** Simulator (or part-task trainer) combining 3D printed technology with other innovative approach to simulate not only the shape of the anatomical structure for surgery but also the observable effect following the simulated surgical procedure (e.g., see “red water” coming out when cutting on simulated skin, hear sound of air leaks when cutting on cricothyroid membrane… etc)
- **Full-motion fidelity:** beyond individual skills training, usually in teams and on either mixed mode (using 2 or above training modalities) or hybrid mode (combining simulators with manikin or simulated patients during the scenario)
