# Supplementary material for: Expanding scope of Kirkpatrick model from training effectiveness review to evidence-informed prioritization management for cricothyroidotomy simulation
Source: Heliyon. 2023 Jul 25;9(8):e18268. doi: 10.1016/j.heliyon.2023.e18268 (PMC10407669; doi:10.1016/j.heliyon.2023.e18268)
Supplement: Multimedia component 3 [file mmc3.pdf]

Appendix 3. Standardized Training Curriculum for Surgical Cricothyroidotomy

Professional Standards

This program was designed in compliance with international standards of emergency airway management:

1. Canadian Airway Focus Group (CAFG) guidelines 2013, suggested that all members involved in trauma management should be trained their skills of Cricothyroidotomy (Law et al., 2013).
2. Difficult Airway Society (DAS) 2015 guidelines for "Technique for scalpel Cricothyroidotomy" emphasized importance of both technical skills and skills concerning human factors (e.g., teamwork and leadership, communication, situation awareness) for reinforcing and retraining skills. (Frerk et al., 2015).

Training Objectives

Participants were able to i) understand the situation of cannot-intubate-cannot-oxygenate (CICO) and standard emergency surgical airway procedure (Cricothyroidotomy) with international guideline and ii) perform bougie-assisted surgical Cricothyroidotomy and maintain ventilation for patients within 2 minutes.

Procedures of Cricothyroidotomy Training

During registration process, participants were provided with information letter and consent form under assistance of research officer. After reading them thoroughly without further questions, they would give e-signature on digital consent form under witness of designated staff. Participants underwent the training cording to the standard rundown.

Rundown of Procedural Simulation Training for Cricothyroidotomy

Flow	Duration	Content/ Remarks
1. Briefing and Familiarization	5 mins	- Training objectives<br>- Familiarization of training environment (e.g., simulator, equipment)
2. Introduction	5 mins	i) Cannot Intubate Cannot Oxygenate (CICO) condition<br>ii) Anatomical physiology of human neck<br>iii) International standard: 2013 Canadian Airway Focus Group (CAFG) and 2015 Difficult Airway Society (DAS)
3. Live Demonstration by instructor	10 mins	with verbal commentary of standard procedure of bougie-assisted Cricothyroidotomy
4. One-off Practice and Skill Assessment of Cricothyroidotomy	20 mins	within 2 minutes under observation by instructor, one-by-one<br>- Step 1: Midline longitudinal incision over skin<br>- Step 2: Transverse incision through the membrane<br>- Step 3: Endotracheal tube insertion with or without a bougie<br>- Step 4: Bagging after connecting Ambu-bag with endotracheal tube
5. Debriefing	5 mins	Review simulated procedure with instructor
6. Wrap-up	5 mins	Recap with take-way message
7. Evaluation	10 mins	Completion of Questionnaire of Usability of Simulator and Training Evaluation via QR code scanning
