# Supplementary material for: Expanding scope of Kirkpatrick model from training effectiveness review to evidence-informed prioritization management for cricothyroidotomy simulation
Source: Heliyon. 2023 Jul 25;9(8):e18268. doi: 10.1016/j.heliyon.2023.e18268 (PMC10407669; doi:10.1016/j.heliyon.2023.e18268)
Supplement: Multimedia component 4 [file mmc4.pdf]

Supplementary 1. Scales of Emergency Surgical Airway Simulator (SESAS-17)

Scales of Emergency Surgical Airway Simulator (SESAS-17)

	**Please circle the appropriate rating on question #1 to #17**	**Strongly Disagree ▼**	**Disagree ▼**	**Neutral ▼**	**Agree ▼**	**Strongly Agree ▼**
01.	The airway model is well featured with reference to real human anatomy.	1	2	3	4	5
02.	Tactile sensation on bagging is enhanced. It feels like bagging on a real patient.	1	2	3	4	5
03.	The model enables clinicians to mimic the whole procedure of surgical cricothyroidotomy.	1	2	3	4	5
04.	The effect of air leak upon incision of cricothyroid membrane looks real.	1	2	3	4	5
05.	The effect of bleeding after incision of skin looks real.	1	2	3	4	5
06.	On bagging after successful intubation, chest rise and fogging of ET tube reassure correct position.	1	2	3	4	5
07.	The model is useful in knowledge and skills acquisition.	1	2	3	4	5
08.	The model is an effective tool to transfer acquired skills from practice to clinical operation.	1	2	3	4	5
09.	With the model, I am capable of conducting performance assessment for trainees. [For Trainer ONLY]	1	2	3	4	5
10	It is easy to handle the model during practice.	1	2	3	4	5
11.	I feel confident using this model alone without technical support.	1	2	3	4	5
12.	I am overall satisfied with the model.	1	2	3	4	5
13.	I will recommend this simulation tool in cricothyroidotomy training to my colleagues.	1	2	3	4	5
14.	I prefer the airway model to pig larynx in training.	1	2	3	4	5
15.	I prefer the new model to rigid 3D printed model.	1	2	3	4	5
16.	The model is safe to use with minimal risk of infection.	1	2	3	4	5
17.	This model has low restriction in use. (e.g., I can practice the procedure out of wet lab)	1	2	3	4	5
18.	Please apply "✓" if you have experience in using items below: ☐ Pig larynx-trachea model ☐ Basic 3D printed larynx-trachea model					
19.	Other comments on the model: ______________________					
20.	Suggestions for improvement: ______________________					

This is the end of SESAS-17. Thank you.
